# Nano-Sampling and Reporter Tools to Study Metabolic Regulation in Zebrafish

**DOI:** 10.3389/fcell.2019.00015

**Published:** 2019-02-19

**Authors:** Thomas Dickmeis, Yi Feng, Maria Caterina Mione, Nikolay Ninov, Massimo Santoro, Herman P. Spaink, Philipp Gut

**Affiliations:** ^1^Institute of Toxicology and Genetics, Karlsruhe Institute of Technology, Eggenstein-Leopoldshafen, Germany; ^2^Centre for Inflammation Research, Queen’s Medical Research Institute, The University of Edinburgh, Edinburgh, Scotland; ^3^Centre for Integrative Biology, University of Trento, Trento, Italy; ^4^DFG-Center for Regenerative Therapies Dresden, Cluster of Excellence, Technische Universität Dresden, Dresden, Germany; ^5^Paul Langerhans Institute Dresden, Helmholtz Zentrum München, Faculty of Medicine, University Hospital Carl Gustav Carus, Technische Universität Dresden, Dresden, Germany; ^6^German Center for Diabetes Research (DZD e.V.), Neuherberg, Germany; ^7^Department of Biology, University of Padova, Padua, Italy; ^8^Institute of Biology Leiden, Leiden University, Leiden, Netherlands; ^9^Nestlé Research, EPFL Innovation Park, Lausanne, Switzerland

**Keywords:** zebrafish, metabolomics, fluorescent reporter, nano sampling, nano scaling, live imaging, beta-cell, diabetes

## Abstract

In the past years, evidence has emerged that hallmarks of human metabolic disorders can be recapitulated in zebrafish using genetic, pharmacological or dietary interventions. An advantage of modeling metabolic diseases in zebrafish compared to other “lower organisms” is the presence of a vertebrate body plan providing the possibility to study the tissue-intrinsic processes preceding the loss of metabolic homeostasis. While the small size of zebrafish is advantageous in many aspects, it also has shortcomings such as the difficulty to obtain sufficient amounts for biochemical analyses in response to metabolic challenges. A workshop at the European Zebrafish Principal Investigator meeting in Trento, Italy, was dedicated to discuss the advantages and disadvantages of zebrafish to study metabolic disorders. This perspective article by the participants highlights strategies to achieve improved tissue-resolution for read-outs using “nano-sampling” approaches for metabolomics as well as live imaging of zebrafish expressing fluorescent reporter tools that inform on cellular or subcellular metabolic processes. We provide several examples, including the use of reporter tools to study the heterogeneity of pancreatic beta-cells within their tissue environment. While limitations exist, we believe that with the advent of new technologies and more labs developing methods that can be applied to minimal amounts of tissue or single cells, zebrafish will further increase their utility to study energy metabolism.

## Introduction

Zebrafish (*Danio rerio*) have evolved from being a model organism primarily used for studies of vertebrate development to a widely applied research tool, including its use in behavioral research, genetics, physiology, disease modeling, toxicology, and drug discovery (Lieschke and Currie, 2007; Kalueff et al., 2013; MacRae and Peterson, 2015; Gut et al., 2017). The growing use of zebrafish is based on the presence of a vertebrate body plan and observations that tissue-specific physiological processes are surprisingly similar between zebrafish and humans (Schlegel and Gut, 2015; Gut et al., 2017). The conservation of the core mechanisms has led to the development of genetic, dietary and pharmacological models to study the principles of energy metabolism under physiological and disease conditions (Santoro, 2014; Gut et al., 2017).

Metabolic diseases are systemic disorders driven by the failure of single or multiple tissues to maintain homeostasis. Loss of metabolic homeostasis occurs when specialized tissues have lost their reserve capacity to react to daily metabolic challenges. For example, the diagnosis of diabetes is preceded by years of compensation during which a reduced number of functional beta cells maintains normoglycemia after meal intake. With further decline of beta-cell function during persistent metabolic stress, a minimum threshold of functional beta-cell mass is reached, causing elevated fasting and post-prandial glucose excursions and ultimately leading to diabetes (Chen C. et al., 2017). Challenging this reserve capacity to study the biochemical processes of cells that protect or accelerate functional responses to metabolic stressors are a cornerstone of metabolic research (Tschop et al., 2012). Due to their small body size, many of the challenges that are routinely done in larger laboratory animals can be difficult to implement in zebrafish. A workshop on metabolism at the 5th European Zebrafish Principal Investigator meeting (March 20–23, 2018 in Trento^1^) was dedicated to discuss novel solutions to remaining obstacles that prevent a wider and more impactful use of zebrafish for metabolic studies. As a result of this discussion, we summarize recent progress in “nano-sampling” approaches for metabolomics studies that provide possibilities to quantify metabolites with tissue-resolution in zebrafish larvae. Furthermore, we outline strategies using reporter tools and live imaging to study heterogeneity of cellular function within the tissue environment, an avenue that holds great promise to investigate the early cellular events leading to metabolic disease with clear advantages of using translucent zebrafish.

## Recent Advances in Nano-Sampling for Metabolomics

Metabolomics technologies have facilitated systematic studies of energy substrates and their intermediates in response to different metabolic states, as well as the contribution of pathologically elevated metabolites to disease onset and progression. Over the past years, metabolomics methods have also been increasingly applied to zebrafish. Typically, samples from pooled embryos/larvae and from adult tissues have been analyzed by nuclear magnetic resonance (NMR) spectroscopy as well as chromatographic or mass spectrometric (MS) methods (Ong et al., 2009; Papan and Chen, 2009; Soanes et al., 2011). [For recent examples by the workshop participants, see (Chatzopoulou et al., 2015; Martano et al., 2015; Weger et al., 2016) and below]. Targeted MS methods can reduce the amount of required material to as few as five embryos at 3 dpf (Kantae et al., 2016), but despite these efforts data interpretation remains challenging considering that metabolites are determined from pooled tissues. Efforts to increase tissue resolution have been made by applying manual microdissection, for example to determine the specific lipid composition of the embryo proper vs. the composition of yolk lipids at different stages, thereby separating two groups of tissues, but still using material from 15 embryos per sample (Fraher et al., 2016). To improve tissue resolution with minimal input material, microneedle sampling has been used to take yolk samples from single embryos for the quantification of drug uptake by targeted UPLC-MS (Ordas et al., 2015). Recently it also has become possible to draw blood from larvae: small drops could be obtained from the posterior cardinal vein at 5 dpf using a microneedle in conjunction with imaging to calculate the sample volume (van Wijk et al., 2018). Using this method the blood concentration, distribution volume and clearance of paracetamol was estimated in response to exposure from the water. Further optimization is required to compare blood sampling from different anatomical locations and to provide additional proof-of-concept examples beyond paracetamol. The determination of pharmacokinetic properties of a small molecule in a zebrafish larvae at nano-scale is a step forward in making zebrafish a suitable complementary model for drug discovery and development.

In another proof-of-concept study *Xenopus* embryos were applied for nano-sampling using a microprobe single cell CE-ESI-MS technique, which could determine about 70 metabolites from single blastomeres from the 32 cell stage (Onjiko et al., 2017). As the sample volumes used in these microneedle-based approaches are similar between zebrafish [20–200 nL; whole 3 dpf larva ∼290 nL (Kantae et al., 2016)] and *Xenopus* (10–15 nL), the capillary sampling method might also allow for untargeted metabolomics in the zebrafish.

Recent technical advances in single cell metabolomics with cultured cells demonstrate the feasibility of reaching cellular resolution also for cells smaller than early embryonic blastomeres with subcellular sampling on the horizon (Esaki and Masujima, 2015); reviewed in Armbrecht and Dittrich (2017), Yang et al. (2017), Qi et al. (2018). Although challenging, microdissections using microneedles or capillaries on tissues from zebrafish will be ideally suited for single cell metabolomics facilitated by the rich resource of reporter transgenic lines for the identification of embryonic and larval anatomical structures.

An alternative approach to nano-sampling is mass spectrometry imaging, which may achieve even higher spatial resolution and give snapshots of *in situ* metabolite distribution. For example, Dueñas et al. (2017) used MALDI imaging to map phospholipid distributions in early zebrafish embryos (up to 16 cell stage) at about 10 μm resolution. However, a downside of MALDI imaging is that it requires cryosectioning of the embryos and has an analytical bias for lipids (Baker et al., 2017; Emara et al., 2017). Secondary Ion Mass Spectrometry (SIMS) achieves higher resolutions than MALDI imaging, but is equally limited to fixed samples (Passarelli and Ewing, 2013; Armbrecht and Dittrich, 2017).

Toward a better understanding of metabolite dynamics, continuous recording of metabolome changes *in vivo* will be required. *In vivo* Nuclear Magnetic Resonance (NMR) spectroscopy is a promising approach for such metabolic monitoring, and a few pioneering studies have followed metabolite changes during development and in response to hypoxia or herbicide exposure in medaka embryos (Viant et al., 2002, 2006; Pincetich et al., 2005). As spectroscopic analysis can be combined with magnetic resonance (MR) imaging, also spatial information on metabolite distribution is accessible to these techniques. For example, Kabli et al. (2009) recorded high resolution localized MR spectra from live adult zebrafish brains with a voxel size of 1.5 mm^3^ and could detect several amino acids and other metabolites. Further improvements of the instruments are likely to increase both metabolite and spatial resolution as well as sensitivity of these methods.

Another strategy to examine metabolism beyond steady state levels is the application of tracer technologies to assess flux rates through different pathways. Mugoni et al. (2013a,b) used ^13^C isotope labeling to study prenyl lipid metabolism in zebrafish embryos, showing reduced Coenzyme Q10 and Q9 synthesis based on HPLC analysis of extracts from 25 embryos mutant for *UbiA-domain containing protein 1* (*ubiad1*) and their wild-type siblings. Combining such tracer studies with the cellular and sub-cellular analysis methods currently being developed should provide unprecedented insight into metabolic pathways and their (patho-)physiological changes *in vivo*. Table 1 summarizes methods that are relevant for nano-sampling strategies in zebrafish.

**Table 1:** Nano-sampling approaches for metabolomics.

Method	Tissue	Description	Amount of material used per sample	References
Manual microdissection	Yolk and embryo proper	Separation of yolk and embryo body with forceps and fine scalpel	Pooled tissues from 15 embryos	Fraher et al., 2016
Microneedle sampling	Yolk, blood	Puncturing and suction of yolk or larval vasculature with glass capillary	Yolk: 50 nL (range 20–200 nL) from 1 embryo Blood: samples pooled from 15–35 individual larvae	Ordas et al., 2015
Mass spectrometry imaging	Early embryos (1–16 cell stage)	Matrix assisted laser desorption/ionization (MALDI) mass spectrometric imaging of phospholipids on cryosections	Cryosections of 1 embryo, spatial resolution 10 μm	Dueñas et al., 2017
*In vivo* magnetic resonance microscopy (MRM)/magnetic resonance spectroscopy (MRS)	Adult brain	Live MRM/MRS of adult fish in flowthrough chamber of microimaging probe	1 adult fish, voxel size 1.5 mm^3^	Kabli et al., 2009


Looking forward, key applications for metabolomics studies in zebrafish include the investigation of cancer metabolism; metabolic reprogramming is a hallmark described as an intrinsic property of cancer and is based on the observation that proliferating cells require a large amount of nutrients, energy, and biosynthetic activity to produce the macromolecular components of the newly generated cells. The zebrafish, with its large collection of genetic models of cancers and the popular transplantation assays, represents the ideal model system for analysis of metabolism during cancer progression (White et al., 2013). While the tools for studying changes in metabolism *in vivo* are being developed, a number of studies have already revealed altered metabolism in a zebrafish model of glioma progression, including changes in glycolytic rate as well as lipid and nucleotide metabolism (Bräutigam et al., 2016; Tan et al., 2016; Zhang M. et al., 2018). Known oncogenes have been reported to rewire metabolic pathways in zebrafish. For example, Yap was found to reprogram glutamine metabolism to increase nucleotide biosynthesis in a zebrafish model of liver hyperplasia (Cox et al., 2016).

To provide a framework to compare metabolic changes in response to reprogrammed metabolic pathways between mammalian and fish metabolism, a metabolic network model (MetaFishNet^2^) has been generated (Li et al., 2010) and refined recently (Bekaert, 2012). Metabolic network models integrate genetic, epigenetic and metabolic information and allow predictions of cancer type-specific metabolic pathways, drug targets and therapeutic strategies, and have been constructed for a number of organisms and tissues [reviewed in Masoudi-Nejad and Asgari (2015)]. The MetaFishNet model was used to draw a comparison between human and fish metabolic pathways, showing a large overlap, and to analyze gene expression data in a zebrafish liver cancer model (Lam et al., 2006). Several metabolic pathways were predicted to be misregulated in zebrafish liver cancer (Li et al., 2010), and Wnt signaling was found to remodel lipid metabolism in tumors induced by overexpression of the oncogene Ras in hepatocarcinoma cells and zebrafish liver tumors (Yao et al., 2018). Advances in nano-sampling and metabolomics requiring low-input of materials will provide powerful technologies to investigate cancer-type specific metabolic changes in zebrafish cancer models.

## Tissue-Resolution of Metabolic Regulation Using Reporter Tools

**FIGURE 1:**
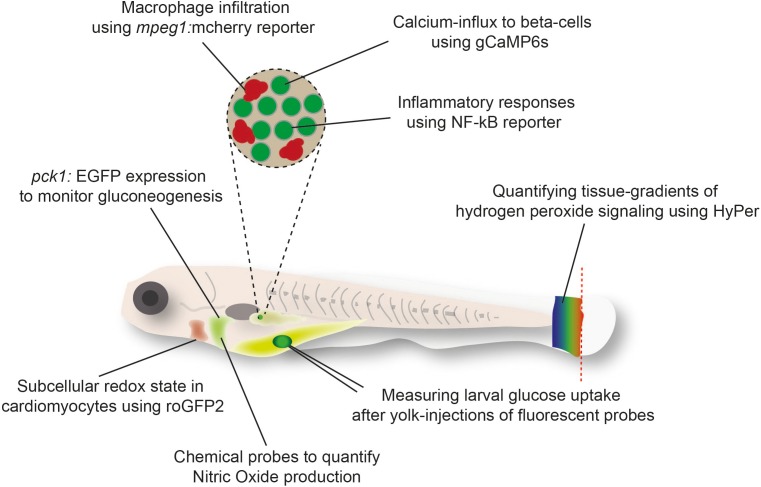
Examples of fluorescent reporter tools used in zebrafish larvae to monitor metabolic responses in cells or tissues.

An alternative to the direct quantification of metabolites in tissues is the indirect visualization of physiological processes (Gut et al., 2013), metabolite ratios (Panieri et al., 2017), or signaling effects of metabolites (Niethammer et al., 2009) in zebrafish using fluorescent probes [Reviewed in Santoro (2014)]. The transparency of zebrafish larvae and amenability to genetic modification has enabled live imaging of these reporter tools in tissues and single cells (Figure 1). Further advantages of these strategies include the possibility to monitor processes continuously and in response to challenges, such as in the background of genetically modified zebrafish, following the treatment with drugs or toxins, or after exposure to tissue damage. For example, the use of genetically encoded H_2_O_2_ sensor HyPer has revealed the critical function of H_2_O_2_ as a chemoattractant released from the wound edge (Niethammer et al., 2009). However, HyPer is also sensitive to changes in pH and therefore requires careful control with pH sensors such as SypHer (Roma et al., 2012; Weller et al., 2014). Further pioneering work has been done using sensor probes for the cellular redox state that are less sensitive to pH, such as recently developed novel transgenic zebrafish lines expressing the metabolic redox biosensors roGFP2-Orp1 and Grx1-roGFP2 (Morgan et al., 2011) in endothelial and myocardial cells (Panieri and Santoro, 2017). These reporters rely on ratio-metric imaging of the sensor for real-time imaging of hydrogen peroxide (H_2_O_2_) levels and the redox potential of glutathione (E_GSH_) in specific subcellular compartments (Panieri and Santoro, 2017). Specifically, imaging these sensors showed higher basal levels of H_2_O_2_ in the mitochondrial matrix than other sub-cellular compartments (Panieri et al., 2017). Similarly, the mitochondrial matrix was characterized by more oxidizing E_GSH_ compared to the cytosol and the nucleus (Panieri et al., 2017). Pharmacologic treatments suggest that the pentose phosphate and glutathione biosynthetic pathways exert a protective antioxidant role *in vivo* in endothelial cells and cardiomyocytes (Panieri et al., 2017).

Similarly, transgenic expression of other ratio-metric fluorescent biosensors for metabolites are on the way. Among those, Perceval HR (Berg et al., 2009) for measuring ATP/ADP ratio and Peredox for measuring NADH-NAD(+) ratio (Hung et al., 2011) are widely used biosensors in mammalian systems. Validation in zebrafish is lacking, but efforts to use them in live larvae are ongoing (unpublished data; YF). Förster Resonance Energy Transfer (FRET) based metabolite reporters are also promising in their application in zebrafish live imaging. Transgenic expression of the lactate FRET reporter. As more genetically encoded metabolite reporters are developed (Jensen et al., 2006; Gruenwald et al., 2012; Luddecke et al., 2017), we envisage that many of these reporters can also be used in zebrafish models to image and quantify metabolism at the cellular and subcellular levels.

In addition to genetically encoded metabolite probes, fluorescently labeled carbon sources such as glucose, lactate, and lipids analogs can be used to trace their uptake into cells *in vivo* in zebrafish embryos [(Marin-Juez et al., 2015; Anderson et al., 2016) and unpublished data; YF]. There is also increasing interest in developing novel fluorescent chemical probes for various metabolites, ions and redox species. Some of these tools have been successfully tested in zebrafish embryos such as a fluorescent sensor to detect Nitric Oxide in liver cells of zebrafish (Zhang et al., 2018a). Other probes include a polymer micelles-based ratio-metric fluorescent probe for hypochlorous acid (HClO) to monitor HClO generation during liver injury *in vivo* in zebrafish embryos (Zhang et al., 2018b).

However, most metabolite sensors are developed and optimized in mammalian tissue culture systems. Additional efforts are required to validate their sensitivity and accuracy in zebrafish embryos, which is particularly the case for FRET probes that often have been optimized to function at 37°C. Once a reliable imaging protocol is established, these sensors will be invaluable tools to monitor dynamic metabolic changes in specific tissues, cells and sub-cellular compartments in physiology and disease conditions. Table 2 summarizes reporter tools that can be used to quantify metabolites and includes information whether these tools have been tested in zebrafish yet. Although these tools will not be able to replace traditional biochemical approaches on sampled tissues, the live observation of metabolites and signaling events *in vivo* can provide invaluable insights into metabolic regulation.

**Table 2:** Selection of reporter tools to quantify metabolites.

Tool	Method	Description	Validated in zebrafish	Reference
Perceval	Fluorescent biosensor	Genetically encoded ratiometric fluorescent reporter for ATP/ADP ratios	No	Berg et al., 2009
GCaMP6s	Fluorescent biosensor	Genetically encoded intensiometric fluorescent reporter for calcium	Yes	Chen J. et al., 2017; Singh et al., 2017; Janjuha et al., 2018a
RoGFP2-Orp1	Fluorescent sensor	Genetically encoded ratiometric fluorescent reporter for H_2_O_2_ detection	Yes	Panieri et al., 2017
Grx1-RoGFP2	Fluorescent sensor	Genetically encoded ratiometric fluorescent reporter for GSH:GSSG redox potential	Yes	Panieri et al., 2017
Cyto-roGFP	Fluorescent biosensor	Genetically encoded ratiometric fluorescent reporter for Redox state (Cytosol)	No	Waypa et al., 2010
Matrix-roGFP	Fluorescent biosensor	Genetically encoded ratiometric fluorescent reporter for Redox state (Mitochondrial Matrix)	No	Waypa et al., 2010
GPD-roGFP	Fluorescent biosensor	Genetically encoded ratiometric fluorescent reporter for Redox state (Mitochondrial Innermembrane space)	No	Waypa et al., 2010
Pyronic	FRET biosensor	Genetically encoded ratiometric fluorescent reporter for pyruvate	No	San Martin et al., 2014
Laconic	FRET biosensor	Genetically encoded ratiometric fluorescent reporter for lactate	No	San Martin et al., 2013
pHRed	Fluorescent biosensor	Genetically encoded intensiometric fluorescent reporter for pH	No	Tantama et al., 2011
Peredox-mCherry	FRET biosensor	Genetically encoded intensiometric fluorescent reporter for NADH/NAD ratio	No	Hung et al., 2011
iNap1	Fluorescent biosensor	Genetically encoded ratiometric fluorescent reporter for NADPH	Yes	Zou et al., 2018
SoNar	Fluorescent biosensor	Genetically encoded fluorescent reporter for NADH	Yes	Zhao et al., 2016; Zou et al., 2018
HyPerRed	Fluorescent biosensor	Genetically encoded intensiometric fluorescent reporter for H_2_O_2_	Yes	Zou et al., 2018


## Reporter Tools to Shed Light Into Cellular Heterogeneity of Beta-Cells

A pertinent example for employing reporter tools to understand the function of individual cells within their tissue-context comes from studies of pancreatic beta-cells. Insulin-secreting beta-cells play a central role in glucose homeostasis, as their loss or malfunction can lead to the onset of diabetes. Beta-cells show a high plasticity in response to metabolic challenges or in pathological conditions, increasing interest in studying beta cell turnover and function at cellular resolution (Ninov et al., 2013; Chen C. et al., 2017). Studies on beta-cell biology in zebrafish so far have mainly used fluorescent reporter lines to study the processes of beta-cell differentiation and regeneration (Prince et al., 2017). These studies have defined the progenitor lineages for beta-cell formation during development and regeneration using genetic lineage-tracing techniques (Hesselson et al., 2009; Wang et al., 2011; Ninov et al., 2013; Delaspre et al., 2015). In addition, they have revealed novel signaling pathways that regulate beta-cell differentiation, proliferation and regeneration (Andersson et al., 2012; Tsuji et al., 2014; Wang et al., 2015) as well as the importance of inter-organ communication (Lu et al., 2016) and the gut microbiota for these processes (Hill et al., 2016).

However, several critical aspects of beta-cell biology that have taken a central stage in the mammalian pancreas field require monitoring of functional read-outs, and await to be examined in zebrafish. For example, the process of maturation of beta-cells toward glucose-stimulated insulin secretion has not been investigated extensively in the zebrafish pancreas. Addressing functionality is important as recent studies in mice have shown that beta-cell death might not be the primary reason for the loss of functional beta-cells in diabetes. Instead, beta-cells in diabetic conditions lose their identity and undergo a process of dedifferentiation, in which they stop expressing beta-cell markers (Bensellam et al., 2018). Thus, it will be necessary to establish models in zebrafish that recapitulate the dedifferentiation of beta-cells observed in mouse and human islets.

Toward this end, it was recently shown that beta-cells in zebrafish larvae show glucose-stimulated calcium influx and expression of markers of mature beta-cells, opening an avenue to use the zebrafish as a model to address the final step of beta-cell differentiation and maturation (Singh et al., 2017). Specifically, the genetically encoded calcium indicator, GCAMP6s, was expressed under the insulin promoter to quantify influx of calcium into beta-cells. Calcium binds to GCaMP6s and activates a conformational change leading to the emission of green fluorescence. Since calcium influx in beta-cells correlates with insulin secretion (Bergsten et al., 1994), this system makes it possible to visualize the function of beta-cells with single-cell precision. When combined with lineage-tracing of different beta-cell populations, this approach revealed the presence of a functional heterogeneity and a trade-off between proliferative potential and maturity among beta-cells (Singh et al., 2017). Further efforts and new tools will be necessary, however, to visualize the actual release of insulin from zebrafish beta-cells, which remains an outstanding goal in the field.

Moreover, the interactions between the immune system and beta-cells play a critical role in diabetes pathogenesis, yet these processes have not been modeled in the zebrafish pancreas. Implementing models of beta-cell inflammation and auto-immunity would allow one to study how these interactions are controlled in response to metabolic stress and aging (Janjuha et al., 2018b). A recent study applied the zebrafish genetics and transgenic reporter for activated inflammation to reveal the presence of an inflammatory clock that marks the proliferative-decline of beta-cells with age. In this clock, beta-cells that activate inflammatory NF-kB signaling also prematurely upregulate *socs2*, an age-related gene that inhibits their proliferation (Janjuha et al., 2018b). This work suggests that certain aspects of beta-cell biology such as their capacity to proliferate depend on interactions with the islet-resident innate-immune cells. However, it will be necessary to further validate the zebrafish as a model to investigate the complex crosstalk of metabolism, immunity and organ function. In this regard, two recent papers showed that *foxp3* marks regulatory T-cells (Tregs) in zebrafish and that *foxp3* mutants display systemic inflammation, suggesting an involvement of these cells in the maintenance of immune tolerance (Hui et al., 2017; Kasheta et al., 2017). These results recapitulate in part the situation in humans where mutations in *FOXP3* predispose to multi-organ autoimmunity (Sugimoto et al., 2017). In the future, it will be important to investigate whether aberrant selection of immune cells during T-cell maturation or prolonged exposure to self-antigens in combination with genetic and environmental risk factors can be applied to model certain aspects of autoimmune diseases such as type 1 diabetes in zebrafish. The repertoire of zebrafish immune cells is not fully understood and one needs to carefully consider differences in immune cell and cytokine profiles between zebrafish and mammals. However, models are emerging that can be used to monitor T-cell development and migration within their niche (Tian et al., 2017; Aghaallaei and Bajoghli, 2018), and will help to further characterize the zebrafish immune repertoire.

Being able to assess beta-cell activity under metabolic and inflammatory stress is critical to identify small molecules that prevent the loss of its function in diabetes. We propose that some of the above-mentioned tools allowing to quantify the rate of ROS production or the metabolic state of cells can be applied to beta-cells as well. These tools, in conjunction with small molecule screening, can facilitate the discovery of novel therapeutic interventions that intervene at different levels in the cascade responsible for beta-cell stress and dysfunction.

## Conclusion and Future Approaches

Progress has been made to exploit the advantages of zebrafish for studying the control of energy metabolism at tissue, cellular and subcellular resolutions. Achieving this level of resolution is critical considering the specialization of metabolically active tissues that often show different, and in some cases even opposite, homeostatic responses to metabolic challenges. Performing whole-larval transcriptomics metabolomics or proteomics analyses therefore provides limited information. The community should apply nano- or micro-sampling approaches wherever possible, facilitated by an active exchange of protocols and access to state-of-the art technologies. The same is the case for the use of reporter tools that often require experience and an optimized set-up, but once implemented provide powerful technologies to perform metabolic studies within the context of a live organism in physiological or pathological states.

## Author Contributions

All authors listed have made a substantial, direct and intellectual contribution to the work, and approved it for publication.

## Conflict of Interest Statement

PG is an employee of Nestlé Research. The remaining authors declare that the research was conducted in the absence of any commercial or financial relationships that could be construed as a potential conflict of interest.

## Footnotes

1 http://events.unitn.it/en/ezpm2018

2 http://metafishnet.appspot.com
